# Higher Nodal expression is often associated with poorer survival in patients diagnosed with melanoma and treated with anti-PD1 therapy

**DOI:** 10.3389/pore.2024.1611889

**Published:** 2024-09-23

**Authors:** Philippe D. Gascard, Xianhong Wang, Mehdi Nosrati, Kevin B. Kim, Mohammed Kashani-Sabet, Thea D. Tlsty, Stanley P. Leong, Mary J. C. Hendrix

**Affiliations:** ^1^ Department of Pathology, University of California San Francisco, San Francisco, CA, United States; ^2^ California Pacific Medical Center, Center for Melanoma Research and Treatment, Sutter Health, San Francisco, CA, United States; ^3^ Department of Biology, Shepherd University, Shepherdstown, WV, United States

**Keywords:** melanoma, nodal, anti-PD1 therapy, immune checkpoint inhibitors, multiplex immunohistochemistry

## Abstract

Advanced melanoma is considered the most aggressive and deadly form of skin cancer whose incidence has been rising over the past three decades. In the absence of treatment, the median overall survival for advanced-stage metastatic disease is less than 6 months. Although most melanomas detected at an early stage can be cured with surgery, a subset of these eventually metastasize. Therefore, a critical need exists to identify unique molecular features that would be predictive of long-term outcome and response to specific therapies. Recent promising therapeutic regimens have included the use of immune checkpoint inhibitors, such as anti-PD1 antibodies. However, the ability to identify responders and non-responders to this therapy remains elusive. To address this challenge at the molecular level, previously our laboratory identified the emergence of a stem cell phenotype associated with advanced melanoma and other aggressive forms of cancer. Underlying this phenotype is the aberrant re-expression of the embryonic morphogen “Nodal”. Particularly noteworthy, we have observed Nodal to remain in advanced tumors of non-responders to standard-of-care therapies (i.e., BRAFi). This pilot study is the first proof-of-principle attempt to predict treatment response survival outcome in a small cohort of melanoma patients receiving anti-PD1 immune checkpoint inhibitor therapy – based on their Nodal expression profile. Using advanced multiplex immunohistochemistry-based digital pathology, the major finding of this preliminary study indicates that higher Nodal expression is often associated with poorer overall survival after anti-PD1 therapy, reaching nearly statistical relevance.

## Introduction

Melanoma is an aggressive type of cancer whose rates have been rising rapidly over the past three decades worldwide. It is estimated that about 97,610 new melanomas will be diagnosed in the United States and that about 7,990 people will die of this disease [1]. However, melanoma mortality rates have declined significantly - by about 5% per year in adults younger than 50% and 3% per year in those 50 and older - since 2011, as a result of diagnostic and treatment advances. Although most melanomas detected at an early stage can be cured with surgery, a small portion of these cancers eventually metastasize. In a continuing effort to advance the field, multiple research teams have concentrated their efforts on identifying unique molecular characteristics associated with primary tumor or sentinel lymph node features that would be predictive of long-term outcome [2–5]. Especially relevant is the recent validation of a 31-gene expression signature [4], which has been developed as a clinical test known as “DecisionDx-Melanoma.” This test allows one to predict the risk of recurrence and metastasis, classifying patients as Class 1A (lowest risk), 1B/2A (increased risk), or 2B (highest risk), and thus inform patient management decisions, resulting in the recommendation of a sentinel lymph node biopsy, additional treatment, or close follow-up after treatment to monitor signs of recurrence.

In recent years, therapeutic regimens relying on the use of immune checkpoint inhibitors (anti- CTLA4, anti-PD1, and anti-LAG3 antibodies) have shown promising results and have therefore become mainstream treatment for advanced melanomas. However, even the success of these latest therapies has been hampered by recurrence and death associated with resistance [6]. Therefore, an urgent clinical need remains to gain new insights into the molecular features underlying the most advanced melanomas to better inform therapeutic strategies and to overcome treatment resistance-associated deaths.

The molecular signature of such aggressive melanomas has come to light in recent years and is associated with the emergence of a plastic, stem cell phenotype [7, 8]. Confirming findings in other highly aggressive cancers, including the re-emergence of embryonic developmental programs and gain in activity of fetal oncogenes – demonstrate that extensive cell plasticity and loss of differentiation and tissue identity are characteristic of such highly lethal cancers. One of the hallmarks of this cancer stem cell phenotype is the pathological re-expression of the TGF-beta superfamily member “Nodal” – occurring in the absence of its embryonic regulator “Lefty” [9], which underlies the progression to advanced disease in melanoma [10, 11]. Also noteworthy is the re-expression of Nodal in other advanced forms of cancer, including aggressive breast cancer [12, 13], prostate, pancreatic, ovarian and colon cancer, in addition to glioblastoma and neuroblastoma [14].

Nodal not only underlies unregulated growth and metastasis, but it is also associated with tumor heterogeneity and resistance to therapy. One example of this was observed in melanoma patients with unresectable stage lll and IV disease -- treated with BRAF inhibitor (BRAFi) therapy. The results showed that BRAFi treatment failed to significantly affect Nodal levels in melanoma tissues from these patients, all of which eventually succumbed to their disease [15].

The management of high-risk cancer patients with heterogeneous tumors is benefiting from an array of increasingly sophisticated assessment technologies. One of these diagnostic tools relies on multiplex immunohistochemistry-based digital pathology, an approach that has gained momentum over the last 10 years [16]. This success has stemmed from a combination of technological advancements, such as: 1) the use of the Tyramide Signal Amplification (TSA) technology that allows greatly increased sensitivity [17]; and 2) the generation of automated staining platforms and imaging and analysis software that mitigates concerns related to procedure inconsistencies and diagnostic discordance.

Given the therapeutic challenges we face with targeting the stem cell phenotype of aggressive tumors described above, we have hypothesized that continued Nodal expression would be detrimental to patient outcome. This pilot study is the first proof-of-principle attempt to predict treatment response and/or survival outcome among melanoma patients -- based on their Nodal expression profile relevant to anti-PD1 immune checkpoint inhibitor therapy. To this end, we first optimized a biomarker panel consisting of Nodal together with a melanoma antigen cocktail used to identify melanoma cancer cells. We found that widespread expression of Nodal (i.e., expression in >45% of melanoma antigen-expressing cells) is associated (with nearly statistical significance) with shorter survival after treatment. Our comparative assessment of patient outcome for specimens presenting with either homogeneous or heterogeneous Nodal spatial expression did not identify patterns associated with either better or worse overall survival outcome with anti-PD1 therapy, likely due to small sample size.

## Methods

### Clinical melanoma samples and patient demographics

The melanoma database at California Pacific Medical Center Research Institute was queried for melanoma patients with sufficient clinical data to assess immunotherapy response. The criteria for inclusion for this analysis were: 1) follow up duration of at least 18 months from the start time of checkpoint inhibitor therapy; and 2) unequivocal response status, either clinical response or early progression to anti-PD1 antibody-based immunotherapy. We identified 25 patients who met these criteria and were included in this analysis. The cohort consisted of 16 males and 9 females all reporting as White/Caucasian and ranging in age from 22.4 to 84.0 years old at the time of diagnosis (median age: 59.0 ± 16.4 years old). Patients were treated with immune checkpoint inhibitors (range: 51–8,616 days post-diagnosis, mean ± SD: 2009 ± 2,199 days) and followed up for treatment response. A responder was defined as a patient whose disease responded to the anti-PD1-containing checkpoint inhibitor therapy with disease control duration of at least 12 months after initializing the treatment. A non-responder (progressor) was defined as a patient whose disease progressed to anti-PD1-containing checkpoint inhibitor therapy within less than 6 months after initializing the treatment. Patients whose disease progressed during 1 year of adjuvant anti-PD1 therapy period were also considered progressors. Patient characteristics, including age at the start of treatment, are summarized in Supplementary File S1. Among the 10 responders, 3 specimens were from regional lymph node metastases, 3 from regional skin/subcutaneous metastases, 1 from distant lymph node metastasis, 2 from distant metastatic organs and 1 from a primary skin site. Among the 15 progressors, 8 specimens were from regional lymph node metastases, 1 from distant lymph node metastasis, 3 from distant metastatic organs and 3 from primary skin sites (Supplementary File S1). For each patient, five-micron thick tissue sections cut from Formalin Fixed Paraffin Embedded (FFPE) melanoma tumor blocks were laid on positively charged Superfrost Plus microscopy slides and processed for Hematoxylin & Eosin staining or multiplex immunohistochemistry (mIHC). Detailed specimen information is provided in Supplementary Files S1, S2.

### Hematoxylin and eosin (H&E) staining

Five-micron thick tissue sections were deparaffinized in two baths of xylene(s) (10 min each), then rehydrated in two baths of 100% ethanol (5 min each), two baths of 95% ethanol (2 min each), and one bath of 70% ethanol (2 min). After a brief wash in distilled water, slides were stained in Mayer’s hematoxylin solution for 8 min. Slides were washed under running warm tap water for 10 min, rinsed in distilled water and then in a bath of 95% ethanol (10 brief dips). Slides were stained in 0.25% eosin Y solution (250 mL 1% Eosin Y solution (Sigma Aldrich Inc.), 750 mL 80% ethanol, and 5 mL concentrated glacial acetic acid) for 1 min. Slides were dehydrated in two baths of 95% ethanol followed by two baths of 100% ethanol (3 min each). Slides were finally cleared in two baths of xylene(s), 5 min each, and then mounted with resin-based mounting medium (Richard-Allan Scientific).

### Multiplex immunohistochemistry (mIHC)

Slides with five-micron thick tissue sections were baked at 60°C overnight, deparaffinized in two baths of xylene(s) (10 min each) and rehydrated in graded ethanols: 100%, 100%, 95%, 85%, 70% (5 min each), and finally in distilled H_2_O. Heat-induced antigen retrieval was carried out in citrate buffer, pH 6.0 (Sigma-Aldrich) at 95°C for 10 min. Endogenous peroxidase and autofluorescence quenching was achieved by incubating slides in PBS containing 4.5% H_2_O_2_ and 10 mM NaOH under a bright light for 45 min. Non-specific antibody binding was blocked using Background Sniper (Biocare Medical). Tissue sections were incubated for 1 h at room temperature (RT) with the Nodal primary antibody in 1% BSA and 30 min in pre-diluted MACH 2 Horseradish peroxidase (HRP) labeled goat anti-mouse micro-polymer secondary antibody (Biocare Medical). The slides were washed in TNT buffer (0.1 M Tris-HCl pH 7.5, 0.15M NaCl, and 0.05% Tween-20), and the FITC signal was developed using Tyramide Signal Amplification (TSA) solution (Akoya Biosciences) for 2 min. Removal of the Nodal antibody complex was achieved by heating in 95°C citrate buffer pH 6.0 for 5 min. Tissue sections were blocked with Background Sniper, incubated for 1h at RT with the melanoma antigen cocktail primary antibody in 1% BSA then for 30 min in pre-diluted MACH 2 conjugated anti-rabbit secondary antibody (Biocare Medical). After washes in TNT buffer, the Cy5 signal was developed using Tyramide Signal Amplification (TSA) solution (Akoya Biosciences) for 7 min. After washes in TNT buffer, nuclei were counterstained with 3 µM DAPI in PBS for 5°min, washed in distilled H_2_O, and mounted with Vectashield HardSet Mounting Medium (Vector Laboratories). Detailed antibody information is provided in Supplementary File S2.

### Imaging

H&E-stained slides were imaged at a ×20 magnification using an Aperio AT2 whole slide scanner (Leica). Analysis was performed using Qupath software (v0.2.0-m3) [18]. Positive pixel count was performed using a threshold for FITC or Cy5 signals based on primary antibody omitted controls (Supplementary Figure S1). Slides stained with the Nodal/melanoma antigen cocktail mIHC module were imaged using a BZ-X800 fluorescence microscope (Keyence). Each multiplex-stained specimen was first scanned at low resolution using ×4 magnification, and then imaged at high resolution at a ×20 magnification - at least three independent ×20 fields were imaged and analyzed to determine positive pixel count. Stitching of overlapping ×20 fields was performed using the BZ-X800 analyzer. Images were prepared as 8-bit merged image files and then as tiff merged image files for quantitative analysis using Image J/Fiji (version 2.1.0/1.53c). Detailed imaging information is provided in Supplementary File S2.

### Quantitative analysis

Images prepared with ImageJ were then analyzed in QuPath as follows: fields were first subjected to single cell detection based upon DAPI nuclear staining using the built-in watershed-based cell segmentation algorithm. Fields were then subjected to segmentation in order to distinguish “epithelium” versus “stroma.” Lastly, positive pixel counting for each dye (classifier) was then performed using fluorescence intensity thresholds defined after analysis of control specimens for which primary antibody had been omitted. Individual classifiers were trained for each marker and combined to create a composite classifier script for automated scoring cells within the annotated regions of interest.

### Statistical analysis

Kaplan-Meier survival analyses were conducted to extract overall survival outcome timelines (calculated from the time of treatment initiation until death or a last follow up) by computing median survival with its 95% confidence interval. Comparative assessments used Fisher’s exact test (GraphPad Prism v10.1.1). Log rank (Mantel-Cox) or Gehan-Breslow-Wilcoxon tests (Chi-square, *p*-value) and corresponding hazard ratios for each comparison are shown in Supplementary File S3. Level of significance used was * < 0.05. Staining, imaging, and scoring of the melanoma tissue specimens were carried out in a blinded manner to ensure generation of unbiased results. Two types of predicted “events” were analyzed: response (or lack of response) to therapy and survival (or death).

## Results

### Design and optimization of a multiplexed immunohistochemistry (mIHC)-based tool to assess Nodal expression in patients diagnosed with melanoma prior to anti-PD1 therapy

**FIGURE 1:**
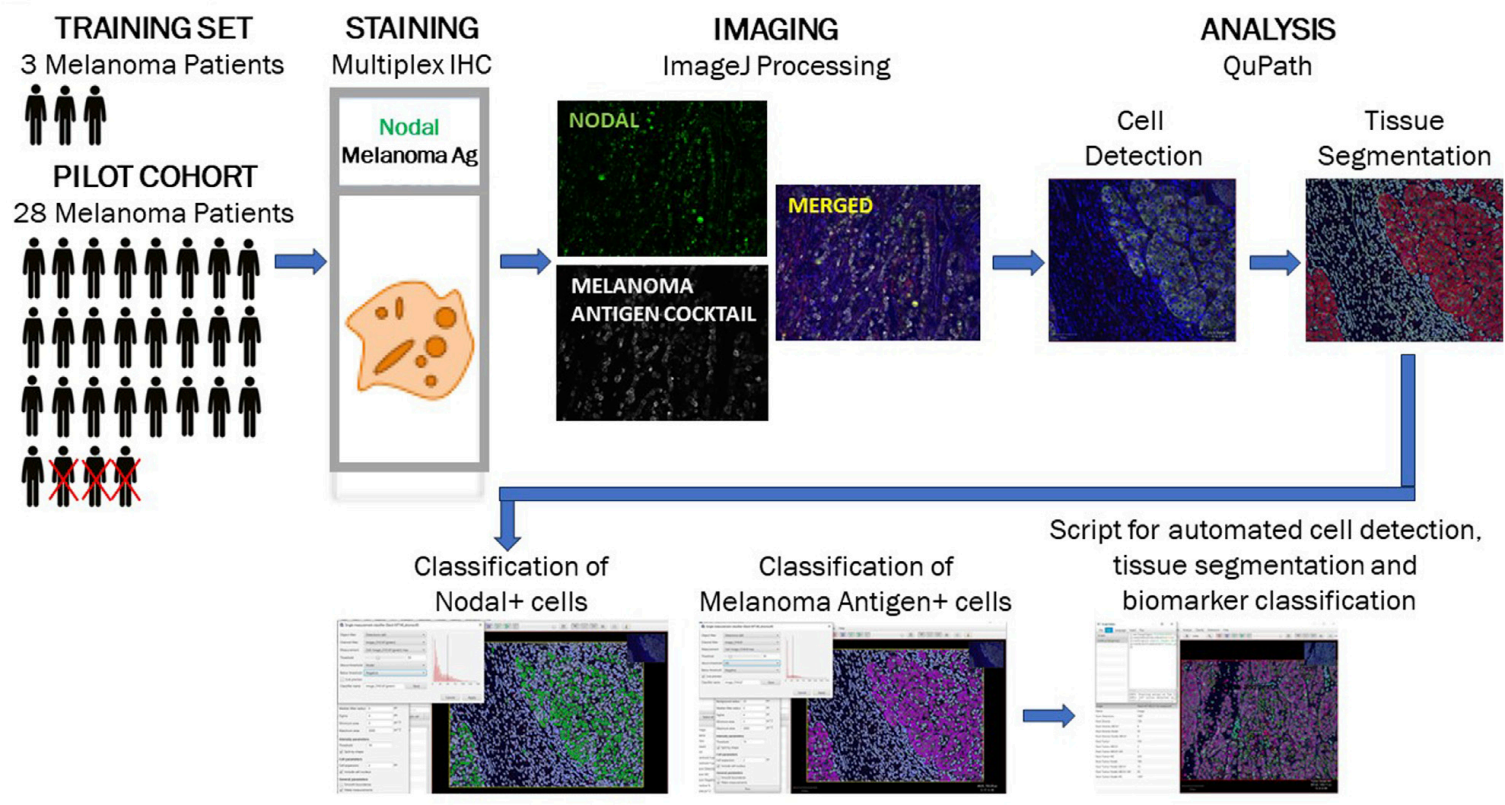
Study workflow for optimization of multiplex immunohistochemistry-based detection of a Nodal/melanoma antigen biomarker module. A training set of specimens collected from 3 melanoma patients was used to design and optimized a mIHC-based detection tool for assessment of Nodal expression in melanoma antigen-positive epithelial cells. The optimized tool was subsequently used to analyze specimens collected from a pilot cohort of 25 patients diagnosed with melanoma prior to treatment with anti-PD1 therapy. The successive steps depicted (immunostaining, imaging and quantitative analysis) are described in detail in the *Methods* section.

**FIGURE 2:**
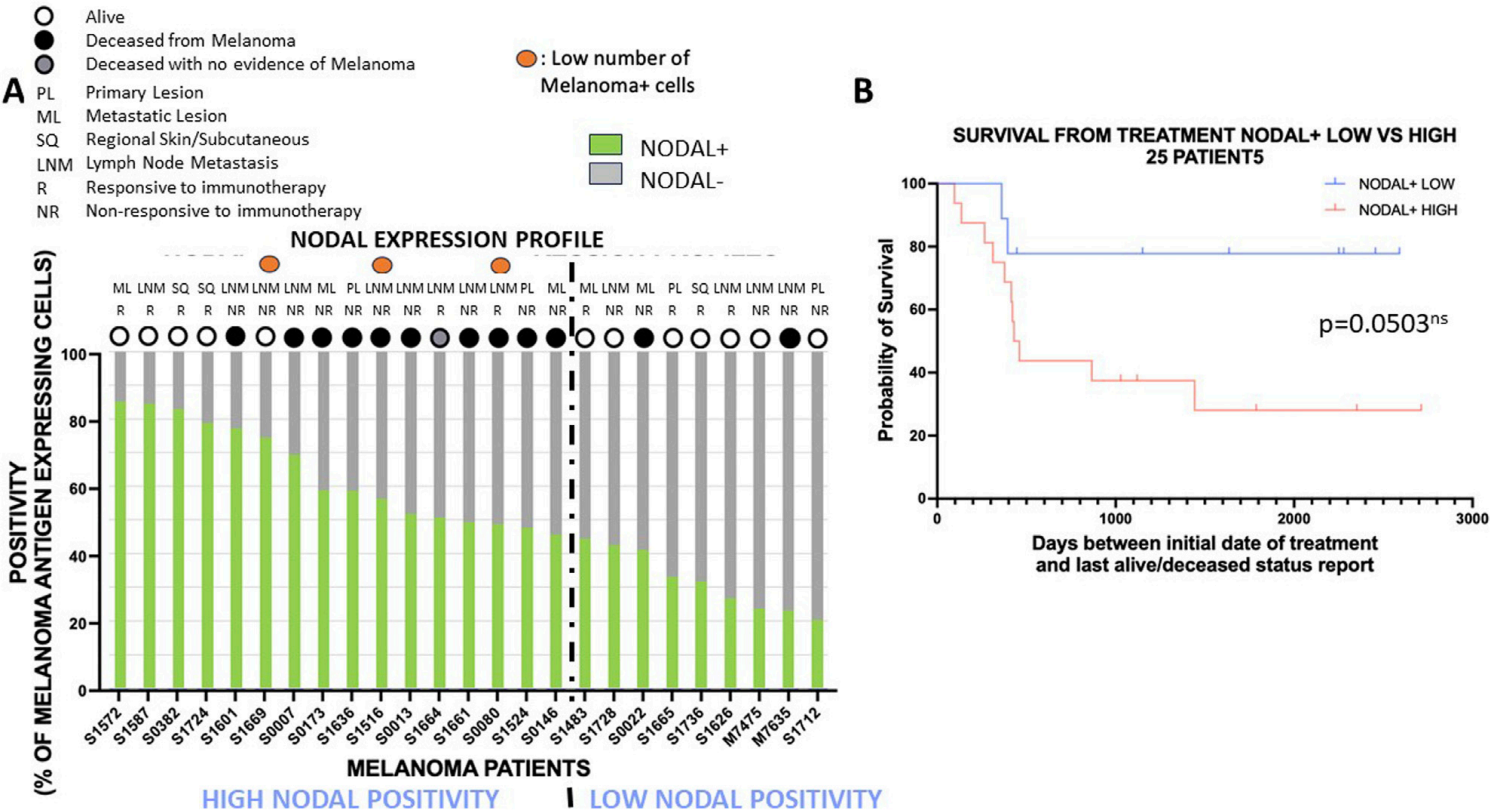
High Nodal positivity is often associated with poor overall survival outcome with anti-PD1 therapy. **(A)**: Expression profiles with percentages of melanoma antigen-positive tumor cells expressing NODAL (green) or not (grey) are shown for each patient. Patients are ranked from the ones with the highest NODAL positivity (left) to the lowest NODAL positivity (right). The dotted line partitions the cohort in two groups: group with the highest NODAL positivity and with the lowest NODAL positivity. Specimen type (PL, primary lesion; ML, metastatic lesion; SQ, Regional Skin/Subcutaneous; LNM, lymph node metastasis), response to therapy (NR, non-responsive; R, responsive), and survival outcome (alive: white circle, deceased: black or grey circles) are mentioned for each patient. **(B)**: Survival analysis for patients with low and high NODAL positivity defined in **(A)**. The high NODAL positivity group had an almost statistically significant shorter overall survival with anti-PD1 therapy than the low NODAL positivity group.

As a first step, we designed and optimized a multiplex immunohistochemistry (mIHC)-based assay to assess expression levels of Nodal in tissue specimens collected from a training set of 3 patients diagnosed with melanoma prior to treatment with anti-PD1 immune checkpoint inhibitors (Methods section; Figure 1). The optimized protocol was then used to stain and analyze specimens available for 25 of the 28 enrolled patients. The analysis allowed us to monitor the number of melanoma antigen-positive cells expressing Nodal or not. Representative mIHC images and downstream imaging and quantitative analysis for two patients -- with either predicted poor or good overall survival outcome -- are detailed in Supplementary Figures S2, S3. The expression profiles for these Melanoma antigen+Nodal+ and Melanoma antigen+Nodal- cell populations are illustrated for the 25 patients in Figure 2. Results were plotted ranking patients according to decreasing Nodal positivity. Results were then contrasted with two endpoints, i. e., subsequent treatment response (responder vs. non-responder) and overall survival outcome (alive vs. deceased).

### Higher Nodal expression is often associated with poorer overall survival outcome with anti-PD1 therapy

We then investigated the potential predictive value of biomarker expression on overall survival outcome in patients who were treated with anti-PD1 immunotherapy. We first assessed the impact of Nodal positivity by assessing the percentage of melanoma antigen-positive tumor cells expressing Nodal or not. The expression patterns across the 25 patients ranked according to NODAL expression identified a group of 16 patients with high NODAL positivity and a group of 9 patients with low NODAL positivity (with ≥45% and <45% of melanoma antigen-expressing tumor cells being positive for Nodal) based upon distinct alive/deceased and treatment response status (Figure 2A). Indeed, the “high NODAL positivity” group showed lower survival, but a similar treatment response compared to the “low NODAL positivity” group [survival: 31.25% (5/16) vs. 77.78% (7/9); treatment response: 37.5% (6/16) vs. 44.44% (4/9), respectively]. A survival analysis showed a nearly statistically significant association (*p* = 0.0503) between NODAL positivity and overall survival outcome (Figure 2B; Supplementary File S3, sheet#5), patients with high NODAL positivity presenting with subsequent shorter overall survival with anti-PD1 therapy.

### Lesion heterogeneity does not predict overall survival outcome with anti-PD1 therapy

Aggressive tumors are often characterized by a high degree of histological and molecular heterogeneity that reflect an extensive loss of tissue differentiation. We therefore conducted a comparative assessment of overall survival outcome for patients with specimens presenting with either homogeneous or heterogeneous Nodal spatial expression (Supplementary File S3, sheet#6). Indeed, we quantified lesion homogeneity/heterogeneity by expressing standard deviation (SD) across the images taken for each specimen. Based on means for NODAL+ positivity (51.99%) for the cohort, we defined heterogeneous specimens as specimens with an SD greater than a 1/3 of the mean, i.e., specimens with NODAL+ SD ≥ 17.33%. Homogeneous specimens were defined as specimens with NODAL+ SD < 17.33%. This allowed us to define two NODAL+ positivity groups with either high (SD range: 18.14%–32.22%; n = 11) or low (SD range: 4.25%–16.65%; n = 14) intra-specimen heterogeneity. Survival analyses showed that lesions with highly heterogeneous NODAL expression were not more likely to be associated with shorter overall survival with the treatment compared to lesions with lower heterogeneous NODAL expression (Supplementary File S3, sheet#6).

### Timeline of treatment post-diagnosis, patient gender or age do not correlate with overall survival outcome with anti-PD1 therapy

Finally, we assessed whether patient profiles would correlate with overall survival outcome post-treatment. As expected, patients not responding to treatment showed a very significant decrease in overall survival outcome, HR = 5.866; *p*-value = 0.0082 (Supplementary File S3, sheet#1). However, since there was a very wide range of time elapsed between diagnosis and implementation of treatment (13–8,616 days), we then compared overall survival outcome for the 12 patients treated early (<1,020 days) post-diagnosis (range: 13–1,017 days; mean ± SD: 350 ± 367 days) versus the 13 patients treated late (>1,020 days) post-diagnosis (range: 1,483–8,616 days; mean ± SD: 3,540 ± 2064 days). A survival analysis did not identify a significant difference in overall survival between these two groups (HR = 0.4349; *p*-value = 0.09), suggesting that patient outcome was not influenced by the time elapsed between diagnosis and implementation of treatment (Supplementary File S3, sheet#2). Survival analyses did not identify any statistically significant association between overall survival outcome post-treatment, including patient gender -- although female patients showed a trend towards improved overall survival (Supplementary File S3, sheet#3), and age at time of diagnosis (Supplementary File S3, sheet#4).

## Discussion

There is a continuing critical need for early detection of patients at high risk for developing cancer as well as early prediction of response to therapy and survival outcome for these patients. This is of particular importance for identifying the most advanced cancers at the earliest stages of development. Cancer progression has been shown to be associated with clinical, but also molecular features, that contribute to the design of customized detection, prevention, and intervention tools. Among the most prominent molecular features in advanced cancers are poor differentiation, i.e., an extensive loss of tissue identity, concomitant with the acquisition of a plastic phenotype. This loss of tissue identity coincides with the re-emergence of embryonic developmental programs underlying a stem cell signature. For example, Nodal is a powerful embryonic morphogen whose expression is quintessential for normal development; however, its aberrant re-expression observed in highly lethal cancers portends unregulated growth and disease progression. Also noteworthy is that Nodal re-expression has been documented with the expression of genes conferring resistance to therapy [12, 13, 19].

In this pilot study, we conducted an assessment on a limited number of clinical samples available to us -- to evaluate the feasibility of using a multiplex immunohistochemistry-based tool to ultimately predict response to therapy and overall survival outcome in patients diagnosed with melanoma and subsequently treated with anti-PD1 immune checkpoint inhibitors -- based upon their Nodal expression profile. This immunotherapy-based regimen has gained momentum in the past decade as it has undoubtedly improved survival outcome in many patients diagnosed with melanoma. However, there remains a notable lack of response to this class of agents and subsequent recurrence in a significant number of patients [6]. Identifying and stratifying patients who will benefit or not from such treatments or who may require combination therapies is therefore of high clinical relevance.

Previously, we observed that higher expression of Nodal correlated with advanced stage disease and resistance to therapy -- in the context of BRAFi therapy in melanoma patients [15] and independently in breast cancer patients receiving standard-of-care therapy [13]. The current study allowed us to extend the BRAFi observations to anti-PD1 therapy in melanoma patients. Since clinical samples were taken before therapy began, our evaluation of the Nodal biomarker -- on a small cohort available to us, was assessed for its predictive value. The clinically relevant observation is that Nodal positivity is associated (with nearly statistical significance) with subsequent poorer overall survival with anti-PD1 therapy. We did not observe any effect of patient gender or age at diagnosis -- on overall survival outcome with the treatment. These observations are in accordance with the results of a large meta-analysis across 801 cancer patients -- representative of nine types of cancer, and 372 respective healthy controls [20]. This analysis concluded that Nodal was frequently expressed in tumors (56.7%) and that higher Nodal expression correlated with tumor size, differentiation degree, and disease progression, but not with patient gender, age or lymphatic metastasis status.

The encouraging preliminary findings that emerged from this proof-of-concept study will require further validation in larger and independent cohorts. With full transparency, we recognize the following limitations of the current investigation: 1) this pilot cohort is small; 2) it includes specimens consisting of regional skin/subcutaneous lesions or lesions isolated from distant organs or lymph nodes; and 3) the timeline between diagnosis and treatment implementation varies widely (13–8,616 days); and 4) anti-PD1 antibody therapies were administered in two different settings: some patients received them in the adjuvant setting, while others were treated in the metastatic setting. In our study, we included adjuvant PD-1 therapies only for early progressors – those whose melanoma progressed during 1 year of the adjuvant immunotherapy, indicating *de novo* resistance to the treatment. Although the responder patients were treated with various agents (nivolumab, pembrolizumab or nivolumab/ipilimumab), these agents have shown the same clinical efficacy (response rates, PFS, 5-year survival rates) in patients with melanoma [21–24]. However, despite all these considerations, the strength of this pilot study includes the demonstrated potential value of using multiplex IHC to assess the expression of specific biomarkers, such as Nodal, for prediction of patient outcome to current therapies. Beyond the scientific and technical advances presented in this study, the data should also provide valuable new tools for clinicians as they determine the most effective treatment for their patients based on molecular biomarkers.

## Data Availability

The raw data supporting the conclusions of this article will be made available by the authors, without undue reservation.

## Supplementary material

SUPPLEMENTARY FILE S1 Detailed patient cohort information. This table provides information related to alive/deceased status, cause of death, gender, race, age at diagnosis, number of days from initial diagnosis to date dead/alive status assessed, number of days from initial date of treatment to date dead/alive status assessed, subtype (pathology) of primary and metastasis diagnosis, treatment and anatomical location/ tissue type of immunostained tissue specimens. For each group (responders vs. non-responders) information includes median age at the start of treatment, gender, stage, serum LDH level, average time from initial diagnosis to immunotherapy initiation (months), average time from immunotherapy initiation to death report/last follow up (months) and type of anti-PD1 immune checkpoint treatment.

SUPPLEMENTARY FILE S2 Antibody and specimen imaging specifications. **Sheet 1**: Description of antibodies used in multiplex immunohistochemistry, including antigen, vendor, catalog number, antibody clonality, animal species in which antibody was raised, working dilution, incubation conditions (time and temperature) and fluorescent dye used for detection. **Sheet 2**: Patient study codes, number of ×20 fields imaged, exposure times for each fluorescent dye (exposure times are identical for all images for a given patient), summary for all ×20 fields combined showing numbers of cells (and percentages in parentheses) including: total number of melanoma antigen-positive tumor epithelial cells and total number of melanoma antigen-positive tumor epithelial cells expressing Nodal.

SUPPLEMENTARY FILE S3 Survival analyses. **Sheet 1**: Comparison of survival between responders and non-responders used as a “quality control” comparison. As expected, there is a highly significant difference between survival for responders (longer survival) and non-responders (shorter survival); HR: 4.443; 95% CI: 1.489-13.26. **Sheet 2**: Impact of timeline between diagnosis and treatment implementation on overall survival outcome with anti-PD1 therapy: no significant difference. **Sheet 3**: Impact of gender on overall survival outcome with anti-PD1 therapy: no significant difference. Note: female patients show longer survival at earlier time points. **Sheet 4**: Impact of age at diagnosis on overall survival outcome with anti-PD1 therapy: no significant difference. A 59-year-old cut off was used as the dividing time point between “young” and “old” since the median age at diagnosis for the 25-patient pilot cohort is 59.0 years old. **Sheet 5**: Impact of NODAL positivity on overall survival outcome with anti-PD1 therapy: close to significant difference. **Sheet 6**: Impact of homogeneity/heterogeneity of NODAL positivity on overall survival outcome with anti-PD1 therapy: no significant difference.

## Abbreviations

BRAF, B-Raf Proto-Oncogene, Serine/Threonine Kinase; BRAFi, B-Raf Proto-Oncogene, Serine/Threonine Kinase inhibitor; BSA, Bovine Serum Albumin; CTLA4, Cytotoxic T-Lymphocyte Associated Protein 4; Cy3, Cyanine 3; Cy5, Cyanine 5; DAPI, 4′,6-diamidino-2-phenylindole; EMT, Epithelial Mesenchymal Transition; FFPE, Formalin Fixed Paraffin Embedded; FITC, Fluorescein Isothiocyanate; HRP, Horseradish Peroxidase; LAG3, Lymphocyte Activation Gene 3; LNM, lymph node metastasis; mIHC, multiplex immunohistochemistry; NaOH, sodium hydroxide; PD1, Programmed Cell Death Protein 1; PFS, Progression-Free Survival; RT, Room Temperature; SQ, subcutaneous; TSA, Tyramide Signal Amplification.
