# Supplementary figures and images for: Higher Nodal expression is often associated with poorer survival in patients diagnosed with melanoma and treated with anti-PD1 therapy
Source: Pathol Oncol Res. 2024 Sep 23;30:1611889. doi: 10.3389/pore.2024.1611889 (PMC11456440; doi:10.3389/pore.2024.1611889)

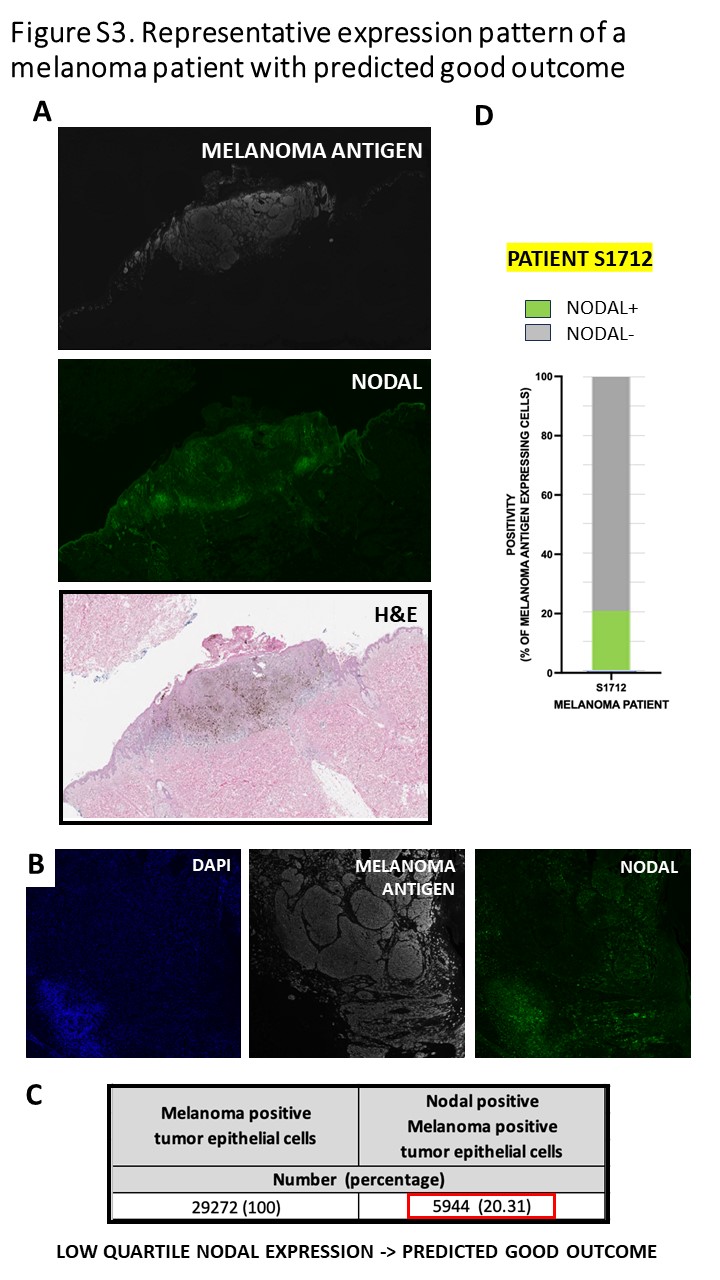

Supplement: Supplementary file 1 [file Image3.JPEG]

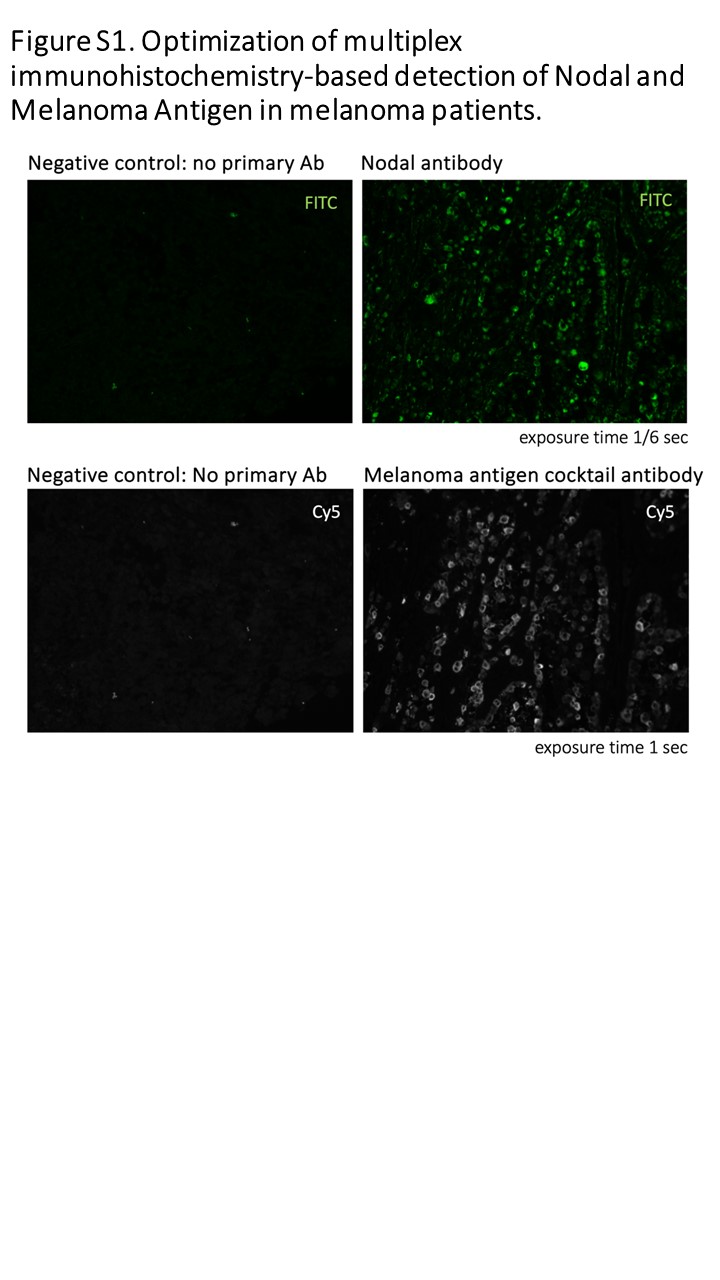

Supplement: Supplementary file 2 [file Image1.JPEG]

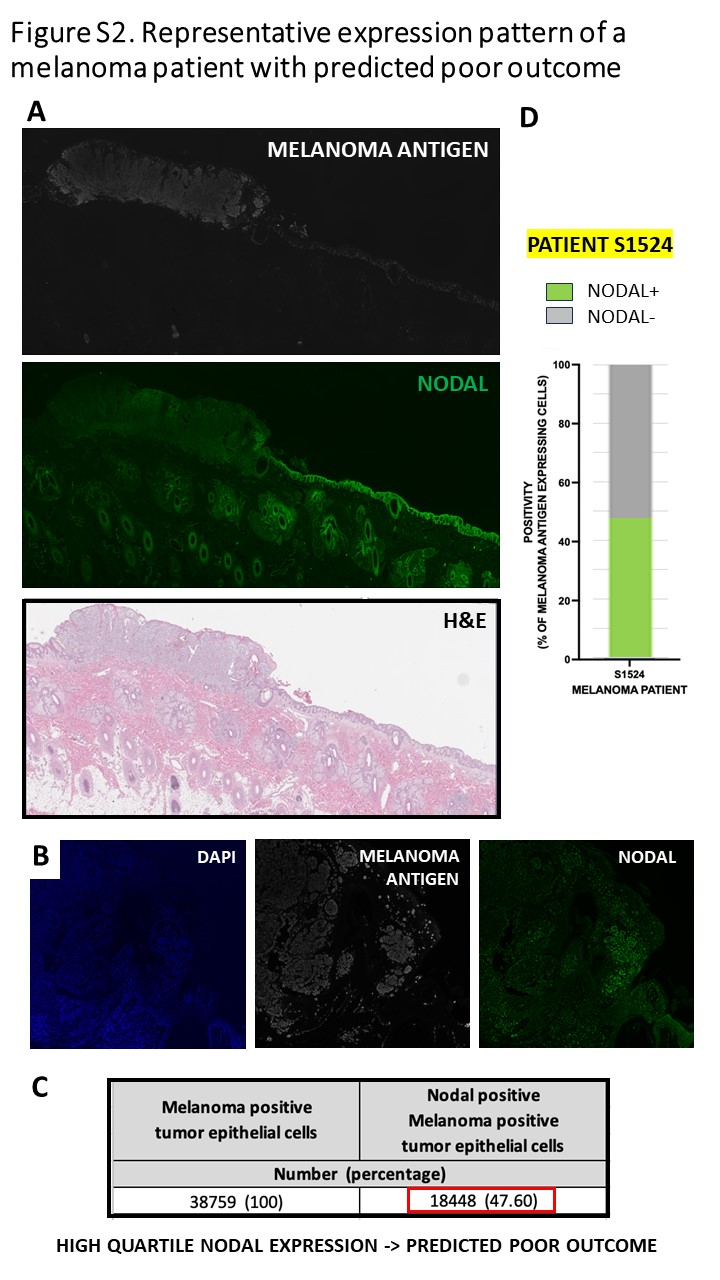

Supplement: Supplementary file 3 [file Image2.JPEG]
